# Oncolytic Viral Therapy and the Immune System: A Double-Edged Sword Against Cancer

**DOI:** 10.3389/fimmu.2018.00866

**Published:** 2018-04-26

**Authors:** Giulia Marelli, Anwen Howells, Nicholas R. Lemoine, Yaohe Wang

**Affiliations:** ^1^Centre for Molecular Oncology, Barts Cancer Institute, Queen Mary University of London, London, United Kingdom; ^2^National Centre for International Research in Cell and Gene Therapy, Sino-British Research Centre for Molecular Oncology, Academy of Medical Sciences, Zhengzhou University, Zhengzhou, China

**Keywords:** oncolytic virus, immunotherapy, host immune response, tumor immunity, cancer-related inflammation, virotherapy, adenovirus, vaccinia virus

## Abstract

Oncolytic viral therapy is a new promising strategy against cancer. Oncolytic viruses (OVs) can replicate in cancer cells but not in normal cells, leading to lysis of the tumor mass. Beside this primary effect, OVs can also stimulate the immune system. Tumors are an immuno-suppressive environment in which the immune system is silenced in order to avoid the immune response against cancer cells. The delivery of OVs into the tumor wakes up the immune system so that it can facilitate a strong and durable response against the tumor itself. Both innate and adaptive immune responses contribute to this process, producing an immune response against tumor antigens and facilitating immunological memory. However, viruses are recognized by the immune system as pathogens and the consequent anti-viral response could represent a big hurdle for OVs. Finding a balance between anti-tumor and anti-viral immunity is, under this new light, a priority for researchers. In this review, we provide an overview of the various ways in which different components of the immune system can be allied with OVs. We have analyzed the different immune responses in order to highlight the new and promising perspectives leading to increased anti-tumor response and decreased immune reaction to the OVs.

## Introduction

A feature of almost all cancers is their ability to escape from the immune system. This process is called immuno-editing and is composed of three different steps: elimination, equilibrium, and escape. During the elimination phase, the immune system recognizes the antigens expressed by tumor cells and eliminates them. If any cells are able to escape from the process of eradication they pass into the second phase in which they modify their antigens to render them unrecognizable by the immune system. In this stage, tumor cells start growing until the mass reaches a considerable volume. This is the third phase of escape, in which the immune system loses control of the tumor which can then spread and become detectable and thus clinically relevant (1). In this landscape, conventional cancer therapies show some limitations. The inability of chemotherapy and radiotherapy to selectively target cancer cells leads to a very high toxicity. Also, the development of chemo-resistance leaves then surgery as the last chance, if available. Another important aspect not to be underestimated, is the lack of conventional therapies able to create long-lasting immunity preventing metastasis and the relapse of cancer.

Cancer immuno-therapy is a promising new strategy to fight cancer and it consists of the activation and arming of the immune system against tumors. There are many different approaches among which, oncolytic virus therapy (OVT) is one of the most encouraging.

As stated in the name, OVT takes advantage of the oncolytic nature of some viruses [oncolytic viruses (OVs)] in order to kill tumor cells. The advantage of these viruses is their ability to infect and replicate in tumor cells without harming normal tissues.

Tumor cells are indeed a good target for OVs. They show a reduction in many of the specific mechanisms used by host cells to respond to viral infection (such as the type I IFN pathway) allowing viruses to replicate successfully in these cells (2). Moreover, advances in genetic engineering have led to the production of viruses lacking the thymidine kinase gene forcing the virus to replicate only in those cells that have an up-regulation of the RAS pathway like cancer cells (3).

Oncolytic therapy is not just a dream. Several viruses have already reached the clinical stages. The best example is given by Talimogene Laherparepvec, known as T-Vec. This is a modified herpex simplex virus (HSV) that has two viral gene deletions and is armed with the human GM-CSF gene. T-Vec has been shown—in a phase II study—to increase the number of tumor-specific CD8^+^ T cells and to reduce the number of regulatory and suppressor T cells (4). Moreover, T-Vec has been tested in a phase III trial in patients with melanoma (5, 6) resulting, in 2015, in the FDA approval for the treatment of melanoma patients with injectable but non-resectable lesions in the skin and lymph nodes (6). Pushed from the good results obtained with T-Vec, over the last few years a variety of OVs have been tested in clinical trials. Safety profiles have reached an excellent standard through modification of OVs to increase specificity and reduce side effects. Despite these promising results, anti-tumor efficacy is still limited (especially when the viruses are used alone) and as a result, new strategies are needed for further improvement of OVT.

In this review, we would like to highlight the promising therapeutic effect of OVT mostly focusing on the ability of OVT to activate the immune system, and how to further improve the anti-tumor efficacy of current OVT by modulating the host immune responses to the viruses and tumor cells.

## Anti-Tumor Effects by OVT

**Figure 1:**
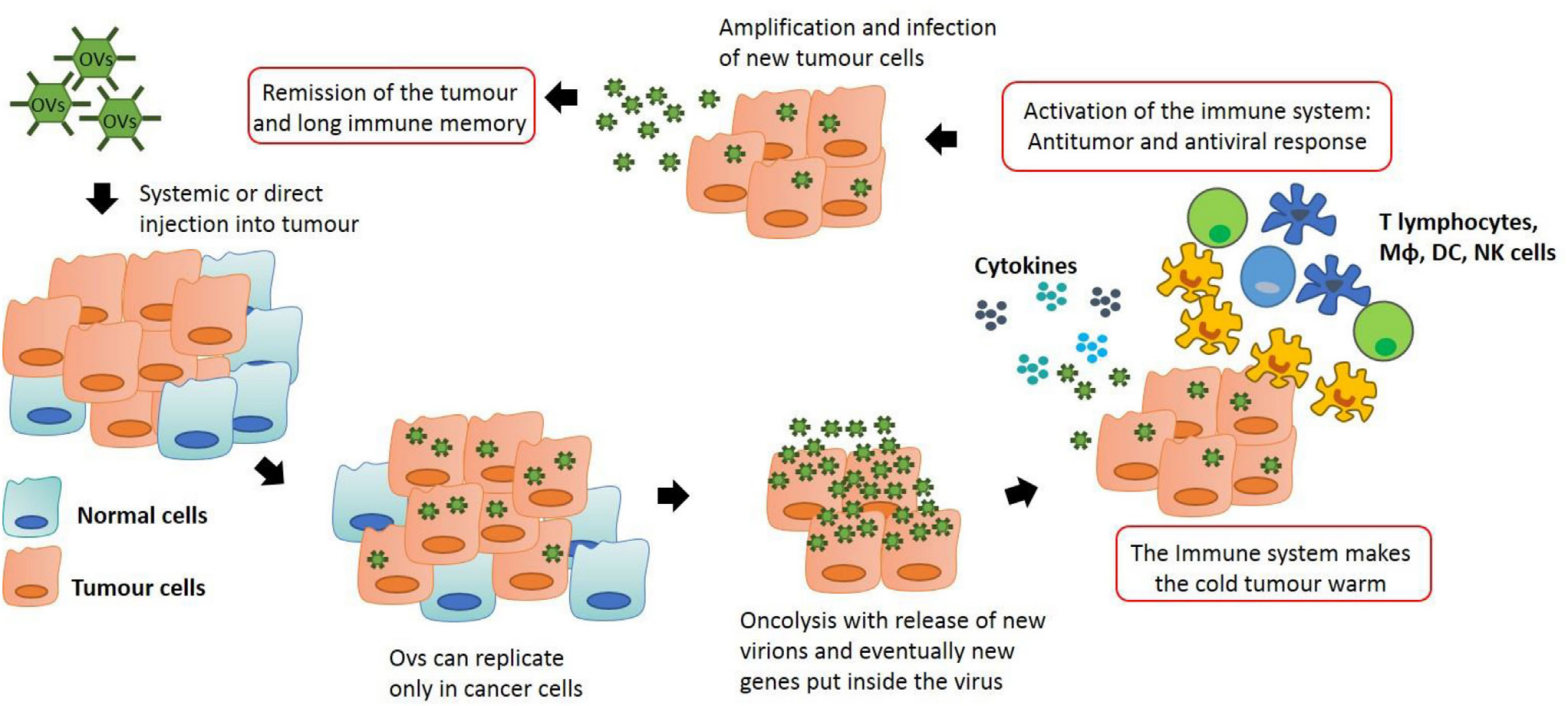
Anti-tumor immunity by oncolytic virus (OV) therapy: OVs can be modified in order to let them to replicate only in transformed cells. This process stimulates the immune system which is recruited into the tumor, skewing the neoplastic mass from an immuno-suppressive environment to an inflammatory site. Macrophages and T lymphocytes are key players in this process, producing cytokines that can recruit other immune cells and actively destroy cancer cells. This action generates an immunological memory that avoids cancer recurrence and synergizes with the oncolytic action of the viruses, potentially leading to tumor remission.

The inhibition of the IFN pathway, the major anti-viral response of the cells, is frequently disfunctioned in cancer cells. As a result, OVs can easily infect the transformed cells and fulfill their function. However, the purpose of oncolytic viral therapy is not only to kill cancer cells but also to activate the immune system, silenced by the tumor microenvironment. In order to achieve this, OVT can act in different ways. OVs are able to create long-lasting memory. As discussed above, transformed cells have the ability to escape from the immune system by mutating their antigens and becoming invisible to leukocytes in a process called immuno-editing. When OVs infect tumor cells, an inflammatory reaction is triggered. This is due to the fact that viruses are able to induce immunogenic cell death (ICD). This process is a particular form of apoptosis in which the death of cancer cells is able to induce an effective anti-tumor response *via* the recruitment and activation of dendritic cells (DCs) and the consequent stimulation of specific T lymphocytes. In ICD, the process of apoptosis is not “sterile” but it triggers the endoplasmic reticulum with the consequent release of some dangerous metabolites called damage-associated molecular patterns (DAMPs). ICD is characterized by the release of three particular molecules that can be classed as DAMPs: calreticulin, ATP, and HMGB1 (7). APCs in the tumor microenvironment recognize these key metabolites and they are able to generate an immune response. Moreover, when OVs infect and destroy cancer cells, tumor associated or/and specific antigens are released into the microenvironment allowing the immune system to recognize them and to generate a response, breaking down the immuno-editing process. As reported by Breitbach et al., this local stimulation of the immune system is able to create a systemic and long-lasting anti-cancer response from immune cells, which also occurs in advanced stage patients (8). In many tumors, the tumor microenvironment is “a cold place” in which the process of immuno-editing has created an immuno-suppressive environment. As reported by Bell’s group, tumor cell infection with OV creates an inflammatory site with the consequent release of cytokines that activate the immune system, making the “cold” tumor “warm” (8). In this way, the primary immune response which can be seen as a negative response triggered against OVs (the activation of the immune system against the virus itself) can create anti-tumor immunological memory with very long-term benefits to protect the host against relapse (Figure 1).

The anti-tumor responses that OVTs can generate are very promising and give hope for new anti-cancer therapies. However, the true potential of OVs cannot be realized until some natural barriers (intrinsic of many tumors) are overcome. Among these, the hardest problems to solve is the large size of the tumor that can deny OVs access to the tumor core along with other physical barriers such as the elevated interstitial pressure and, in case of intravenous delivery, the poor tumor vasculature.

## Anti-Viral Effects by OVT

The battle between anti-tumor and anti-viral immunity exists because triggering the immune system will clear virus and along with it the lytic effect of viral infection, however, anti-tumor immunity will then also be diminished. We need to discover whether or not to focus on engineering a virus that very efficiently kills tumor cells or a virus that stimulates the most robust anti-tumor response.

However, OV infection is the reason why anti-tumor immunity is generated. Upon targeted infection of tumor cells, the immune system detects viral infection and as well as innate immunity, adaptive responses are also triggered. These involve T cells which are primed to lyse cells containing foreign antigens (which usually derive from virus but can also be derived from the cancerous cell itself). This ultimately leads to targeted lysis of not only infected cells but also, the tumor cells themselves. It is therefore important to design OVs that can replicate and spread within tumors quickly to induce the maximal anti-tumor effect before viral clearance (9).

In light of the fact that anti-viral responses may dampen the effect of OVs by clearing virus prematurely, manipulating the anti-viral immune response by blocking antibody responses to the virus means that the virus has extra time to take effect and kill tumor cells through anti-tumoral T-cell responses generated by the presentation of tumor antigens by infected cells (10). Recently, we demonstrated an innovative way to bypass anti-viral immunity, showing that repeated local administration of adenovirus can improve the efficacy of anti-cancer therapy in a Syrian hamster model (9). Li et al. demonstrate that if the virus is injected intratumorally, humoral immunity has no effect on the clearance of the virus which suggests innate immunity and cytotoxic T cells plays a central role. Moreover, they demonstrate that pre-existing immunity against the virus does not affect therapeutic efficacy. As a consequence of this, repeated administration of the virus to the tumor site can trigger a robust immune response against the virus. In this way, a great number of tumor cells are destroyed due to viral infection and the release of several antigens into the tumor microenvironment triggers a huge immune response against the tumor (9).

Anti-viral immunity occurs as the immune system responds to the presence of virus in tumor cells within the body. This attracts various types of immune cell to the site of infection including innate cells (e.g., NK cells) and adaptive cells (e.g., CTL). These immune cells will lead to the destruction of infected cells (i.e., tumor cells) which augments the direct lysis of tumor cells by viral infection itself. This effect can be improved by arming viruses with immune modulatory proteins like cytokines which aid in the attraction of immune components to the tumor site (11).

## How Innate Immune Cells Affect Anti-Viral Therapy

Intravenous injection with OVs can have many positive effects, such as the ability to reach metastatic lesions, but it also exposes the virus to the action of the immune system. Innate immunity is very active in this way. Macrophages are the first line of defense in the host and they naturally work as scavenger cells. They are part of the innate immune system and can recognize pathogens quickly and in a non-specific way in order to initiate an adequate inflammatory response. However, in oncolytic therapy this presents a significant hurdle. Systemic delivery of virus can be affected by macrophage phagocytosis (12) and various studies have also found that after OVT, large amounts of virus are often found in the spleen and liver following capture by mononuclear phagocytes (13, 14). Researchers have developed some strategies in order to overcome this problem. Chemical modification of viral coat proteins is a strategy used to mask the virus and allow it to reach the tumor tissue. To address this topic, some groups have taken advantage of polyethylene glycol (PEG), a hydrophilic, non-immunogenic, and uncharged compound that is able to interact with the biological material to avoid—or at least limit—protein–protein interactions (15). Eto et al. used PEG to coat the adenovirus surface, avoiding its identification by macrophages and prompting the use of this strategy for all applications that require systemic administration (16). Moreover, macrophages recognize antigens *via* scavenger receptors which can be saturated and therefore pre-conditioning macrophages before viral administration is a valid method of temporarily inhibiting macrophage function. Another way to overcome the challenge is to deplete macrophages before administration of the virus (16–18). The best way to achieve this is to use clodronate liposomes. The mechanism of action is very simple: macrophages phagocytose the liposomes and as a result of phospholipase-mediated disruption of the liposomal structure and consequent release of the clodronate, cell death is triggered. This approach can work in order to deliver the virus to its correct site, but can also reduce the anti-tumor efficacy of the therapy. Indeed, as mentioned above, it is important for the anti-tumor therapy that the neoplastic mass is transformed from “cold to warm” in order to re-activate the immune system, kept silent by the tumor itself.

Tumor-associated macrophages (TAMs) are a key component of this process. Macrophages can polarize into pro-inflammatory cells (M1 classical activation) that sustain the Th1 response, or into anti-inflammatory, tissue repairing cells (M2 alternative activation) that sustain the Th2 response and create an immuno-suppressive environment (19). TAMs are mostly M2-like and produce cytokines, such as TGF-β and IL-10, in order to make the microenvironment non-reactive against tumor antigens. Despite this, they can twist their phenotype according to the microenvironment (20). OVT is able to skew the M2-paradigm, activating the immune response against tumor cells. Therefore, it is important not to destroy macrophages but to re-educate them to become powerful weapons. Tan et al. show in their work that TAMs can help the oncolysis of attenuated measles and mumps virus *in vitro* (21). Despite the limitations of *in vitro* work, it opens the field to a new concept. They sustain that viral administration can change the tumor microenvironment making it “warmer,” so making it a site of inflammation. In this context, TAMs (as plastic cells) can change their phenotype from M2-like to M1-like, upregulating their anti-tumor properties. *In vivo*, when the therapeutic virus reaches the neoplastic border and undertakes its lytic effect, it allows the release of many tumor antigens into the tumor environment. In this context, macrophages and DCs recognize the antigens, migrate to the lymph node where they activate T cells that are recruited into the tumor site and can exploit their anti-tumoral functions. In this way, manipulating macrophages to carry vehicle viruses into the tumor and using them as APC, appears to be a very promising strategy to improve the efficacy of OVT.

For these reasons, macrophages are the true double-edged sword of the immune system. It is very important to keep them under control, in order to avoid the clearance of the virus, but at the same time, they represent a potentially useful weapon against cancer due to their ability to “wake up” the immune response.

## How Adaptive Immune Cells are Involved in OVT

Macrophages, DCs and other APCs have the crucial role of activating the adaptive immune response. Lymphocyte activation is slower than the innate immune response but it is more powerful and specific. Lymphocytes recognize specific antigens and start a reaction against them in a pre-determined way. The final aim of immune stimulation during OVT is to activate T lymphocytes against tumor antigens. T cells can recognize tumor peptides and provide long-term immunity. This means that not only will the primary tumor be affected but metastatic sites will as well. Moreover, due to the ability of T cells to generate memory, the patient will be protected even in the case of relapse. For this reason, tumor cells often attempt to become invisible to the immune system in order to avoid activation of lymphocytes. OVT is able to generate a cytokine storm that results in the recruitment of lymphocytes, breaking down the immuno-suppressive environment. Unfortunately, T lymphocytes do not only react against tumor cells but they are also able to initiate a strong and rapid anti-viral response. Managing the balance between anti-viral and anti-tumor activity is a big issue currently under investigation.

Over the last few years, checkpoint pathways have generated a lot of interest. T lymphocytes, as soon as they are activated, start to express checkpoint molecules, such as PD-1 and CTLA/4. During “physiological inflammation” these proteins have the key role of switching off T cells in order to block the aberrant inflammatory response and to avoid the rise of autoimmune disease. Unfortunately, tumors express PD-L1, the ligand of PD-1, in an attempt to switch off the powerful arm of immunity against cancer through the mechanism of immune evasion. For that reason, a lot of effort has been put into developing a way to block these pathways and allow T effector cells to perform their role against the tumor. Ilett et al. demonstrate that the sequential administration of different viruses in combination with an immune checkpoint inhibitor can generate a fully systemic anti-tumor immunity in a model of melanoma (22). They set up a model in which pre-conditioning with GM-CSF prior to administration of Reovirus, allows the OV to initiate tumor killing by potentiating innate immune activation. The result of this treatment is a low and indirect induction of a Th1 response against tumor antigens. The second part of the treatment consists of the administration of VSV-ASMEL, a VSV-c-DNA library of human melanoma cell lines, encoding for a huge number of tumor antigens. This results in the spread of tumor antigens into the tumor. Previous delivery of the GM-CSF/Reovirus had generated a weak T-cell response. CD4^+^ cells recruited into the microenvironment can now come in contact with a huge quantity of tumor antigens and, despite the weak primary response they can now expand and give rise to a strong cytotoxic response. Finally, the administration of anti-PD-1 antibody facilitates a strong and sustained response (22). Obviously, this approach has some limitations such as the fact that, as highlighted by the authors themselves, it is impossible to reach the clinical level with the huge library of tumor antigens that were used. Despite this, they proposed a new model to improve tumor killing by avoiding the initiation of the anti-viral response. But the most significant result has been reached by Ribas et al. The authors performed a phase Ib clinical trials using T-Vec in combination with the anti-PD-1 antibody pembrolizumab. The treatment with anti-PD-1 is able to prolong the effect of cytotoxic T lymphocytes and the main cause of its failure is due to the lack of CD8^+^ cells inside the tumor lesion. Ribas et al. demonstrated that the intratumor injection of T-Vec is able to recruit CD8^+^ lymphocytes into the tumor making them responsive to pembrolizumab. Moreover, the combined treatment is able to increase the circulation of both CD4^+^ and CD8^+^ T cells, proving a systemic and durable response (23). This approach seems to be very effective. Indeed, many new different works are trying to demonstrate the efficacy of the combination of OVs and anti-checkpoint inhibitors. Bourgeois-Daigneault et al. have shown the positive effect of the combined therapy of Maraba rhabdovirus and immune checkpoint blockade in triple-negative breast cancer (24). Analogous to this Samson et al., demonstrated that this approach is worth also in brain cancer. They showed that the intravenous injection of orthoreovirus in high-grade glioma and in brain metastases is able to increase the T-cell infiltration, improving mice survival after later anti-PD-1 treatment (25). Similar results were recently obtained in our group using a different approach. Chard et al. demonstrated the efficacy of VV armed with IL-10. IL-10 is an immuno-suppressive cytokine and they proved that administration simultaneously with the virus inhibits early immune response to infection resulting in a dampening of anti-viral but not anti-tumor immunity (26).

## Strategies to Improve the Induction of Tumor-Specific Immunity by OVT

Oncolytic viral therapy has beneficial effects on the immune system. As described before, it is able to create long-lasting immunological memory thereby avoiding relapse and metastatic spread. It has been identified in certain cases that the anti-tumor immune response is much more important for clearing tumor than is the direct oncolytic effect of the virus.

Recently, mainly thanks to the advances made in genetic engineering, new OVs have been made by the insertion of genes encoding for proteins able to stimulate the immune system. The most well-known virus made in this way is T-Vec—already approved by FDA—an HSV-1 virus modified to carry the human GM-CSF gene that has been demonstrated to increase the number of monocyte-derived DCs and, as a consequence, to increase the activity of cytotoxic CD8^+^ lymphocytes by promoting antigen presentation. Considering these promising therapeutic effects, other viruses such as Vaccinia virus, have been modified to carry this gene.

Based on these encouraging results, other strategies have been developed to include interleukins in order to potentiate the adaptive immune response. Among these, IL-2 has been used to activate T lymphocytes and IL-12, IL-15, and IL-18 have been used to activate both T and NK cells (27–31). Also, other interleukins such as IL-4 have been tested (in adenovirus) but with unsatisfactory results from a safety point of view (32). In addition to cytokines, chemokines have also been used to engineer OVs. The most promising results have been obtained from the insertion of CCL5 and CCL3 in adenovirus. CCL5 can attract T lymphocytes to the tumor, while CCL3 is able to recruit neutrophils into the tumor (33, 34). As discussed, the immune system is activated by OVTs and new strategies are now rising in order to improve the immune response. Insertion of a particular cytokine into the virus can attract a specific immune population into the tumor site, favoring the killing of cancer cells and the breakdown of the immuno-suppressive microenvironment.

One of the promising avenues of OV enhancement is to combine them with the use of T-cell engager molecules in order to stimulate existing T cells to lyse tumor cells by creating a bridge between the two, thus allowing the activation of the cytolytic properties of the T cells present in the tumor. It has been shown that the use of bi-specific T-cell engager molecules can induce T-cell activation even when MHC-I is absent from the surface of the target cell. This feature of T-cell engagers would help to overcome one of the major problems of cancer cells which is immune evasion through downregulation of MHC-I molecules displayed on the surface (35). It has also been shown that the use of bi-specific antibodies that target both cancer cell markers and T cells can lead to long-term anti-tumor immunity which is an important aspect of cancer therapy (36). Using this therapy in combination with OVs has been seen to improve the T-cell killing of tumor cells in various studies (37, 38). This is because in addition to the direct oncolytic effect of the parental virus, the T-cell engager expressing virus can also recruit the existing T cells to fight cancer cells thereby increasing the number of methods in use to eliminate tumor cells.

However, a potential limitation of this therapy is that high levels of Tregs in individual patients can result in lower efficacy as Tregs reduce the level of T-cell killing activity (39). This can be overcome, however, by the removal of Tregs before treatment to eliminate the dampening of the T-cell response by these cells.

Another positive effect of arming OVs with T-cell engagers is the bystander effect demonstrated in specific cases. It has been shown that tumor cells negative for the target molecule of the engager (in this case EGFR) were lysed when in proximity to tumor cells that display the target antigen through T-cell induced bystander cell lysis (40). A phase II clinical trial has been reported for T-cell engager molecules which showed promising initial results but leaves room for improvement in terms of delivery regime in order to reduce the number of patients who need to drop out of the treatment early due to side effects. It would also be worth initiating a study to analyze the effect of T-cell engager delivery by OVs to target tumor cells and potentially reduce side effects.

In parallel to this, our group studied a new elegant strategy to boost the immune response against tumor cells. When a virus is injected, the immune system reacts against it generating an anti-viral response. In some cases, this reaction is too strong and it results in the complete clearance of the virus. In order to overcome this problem, we set up a therapeutic regime in a Syrian Hamster model whereby they were challenged with two different OVs: adenovirus first followed by Vaccinia virus administration (41). In this way, we demonstrated that the immune system reacts against adenovirus leaving the VV able to exploit its anti-tumor function. It was also found that the addition of the second virus can boost the anti-tumor immune response. This occurs as the immune system is focused on developing a specific response against the first virus and is not able to simultaneously inhibit the therapeutic functions of the second. Nevertheless, further studies are needed in order to demonstrate the possible role of innate immunity in this process.

In addition, neutralizing antibodies (NAb) represent another relevant face of immunity that is worth considering. Normally, they recognize the virus, coat it and lead it to be phagocyted by competent cells. However, Adair et al. show that Reovirus administered i.v. can be rapidly cleared in the plasma but not in the cellular fraction of the blood because it is able to form complexes with blood cells. Further work allowed them to discover that the access of Reovirus to tumors can be paradoxically enhanced by the presence of anti-viral NAb which form complexes with Reovirus for uptake, carriage, and delivery to tumors by monocytes in the blood (42, 43). However, normally NAb block the systemic delivery of almost all OVs therefore a deeper analysis and characterization of this process is necessary.

Finally, another therapy used to boost the immune system is named “trojan horse” therapy. In this approach, immune cells are directly infected with the virus *ex vivo*. Studies have revealed increased efficacy with this methodology by which the immune cells are able to directly load the virus into the tumor site (44, 45) retaining the positive effect that OVT has on the immune system. Despite the promising results, there are some limitations to this technique considering that some tissues are not suitable for this therapy. For example, it is not possible to infiltrate the brain with high numbers of molecules and cells.

Intratumoral delivery of the virus can affect the local tumor microenvironment. However, it is not always feasible because of the inaccessibility of the tumor itself. Pancreatic tumors, for example, are very difficult to directly reach. Moreover, local injection of the virus does not take into account potential metastatic lesions. To overcome all of these issues, an alternative to *in situ* delivery is represented by intravenous injection. Intravenous delivery has the potential to reach metastatic lesions as well as the primary tumor even if they are not clinically detectable. Russell et al. (46) demonstrated in a phase I study that a complete and durable response was generated in myeloma patients treated intravenously with an oncolytic Measles virus. Of note, this treatment worked only in those patients without NAb. NAb are one of the major obstacles to intravenous delivery of OVs. However, it has been shown that some viruses, such as Reolysin, are not affected by the presence of nAbs (42). Even if relatively few results have been achieved so far in this direction, a new prospective in the field is now open.

## Potential Risk for Modulating OVT-Mediated Host Immune Response

Whilst oncolytic therapy can be modified to produce the desired effect in a multitude of ways, the outcome of treatment is not always positive. One of the major obstacles to successful oncolytic therapy is the host immune response to viral infection, especially when the virus is administered IV. Indeed, after IV injection, monocytes and macrophages phagocyte the virus, preventing it to reach the tumor site. This response is highly tuned and extremely effective in most cases and therefore, overcoming this hurdle requires careful design of optimal viral gene deletion and insertion combinations. A good study in this direction in represented by Enadenotucirev, a tumor-selective chimeric adenovirus that has been recently used in a phase I clinical trials. Garcia-Carbonero et al. made a study—in collaboration with PsiOxus Therapeutics—injecting the virus both IV and IT in patients with different epithelial tumors. They found no toxicity caused by IV injection. They showed increased levels of some inflammatory cytokines after the infection which rapidly return to the pre-injection levels in 48 h. The authors claim that this is due to a low-dose infection. Their treatment seems able to decrease the cytokines toxicity, allowing the IV treatment (47).

The main side effect of the host immune system is efficient clearance of virus and therefore diminished effect of treatment. In this case, the host immune response to viral infection is such that the virus is cleared from the body before it can have an impact on its target tissue (mostly affects viral treatments which are delivered systemically). In order to combat this, viruses can be delivered within host cells which are extracted, infected, and subsequently replaced. This provides a route into the body which is sheltered from the host immune response.

Another problem posed by the host immune response is hyper activation in response to viral treatment. This problem can be combated by attenuation of the virus in order to allow delivery of a higher dose with fewer side effects than a more virulent strain. Subversion of certain pathways involved in the host immune response can also be used in order to dampen the immuno-pathology whilst still allowing generation of anti-tumor immunity.

We also need to consider the feasibility of delivering oncolytic therapy to vulnerable patients such as the elderly and immuno-compromised. In these cases, it is imperative to ensure that the virus does not infect and cause chronic infection due to lowered immune responses as this could lead to negative side effects and resistance to that particular viral treatment option. On this note, it is also important to consider the suitability of each patient for oncolytic therapy. It has been reported that levels of chronic innate immune stimulation detected in patients correlates with the outcome of oncolytic viral therapy. It was seen in this study that higher levels of chronic immune stimulation in patients before treatment with oncolytic adenovirus correlate with poor prognosis, suggesting that patients with low levels of chronic immune stimulation are more likely to respond positively to oncolytic viral therapy (48).

It is also important to keep in mind that manipulation of viruses by adding cytokines also manipulates the microenvironment. Cytokine treatment requires careful monitoring of concentration. It is important to find a balance between the desired effect and the unwanted side effects that the treatment can create. For example, using PD-1/PD-L1 inhibitor poses the risk of a persistent activation of the immune system and a consequent initiation of autoimmune disease.

Finally, to make sure that research efforts are well spent, we should take in consideration the animal model used. Mice are widely used in research laboratories but sometimes they are not the best model, especially for assessment of OVs. Many OVs are species-specific and cannot efficiently replicate in murine cells without any effects on human cells. This is a particular issue for human-specific adenoviruses that have been reported to have a very poor replication in a great variety of murine tumor cell lines. To avoid this issue, sometimes xenograft models are used in which human tumor cells are implanted into an immuno-compromised recipient. However, this approach does not take in consideration of the complexity of the tumor microenvironment and it is a simplistic solution that cannot be validated for all studies. Tysome et al. propose a different approach, using Syrian hamster as an immuno-competent model. They showed that this model is able to support Human-Adenovirus and Vaccinia virus replication, providing a valid alternative to murine models (41).

## Future Remarks

Oncolytic virus therapy is a very promising new treatment for many different types of cancers. Theoretically, every kind of cancer could be treated by viruses. The main problem of this approach is that the pre-clinical animal models could unfortunately have a very different anti-viral response with respect to humans. Considering this, animal studies should be carefully designed and performed. Another limitation in OVT is represented by the immune system itself in that, as mentioned above, it can start a reaction against the virus. It is important to find a balance between the immune anti-viral response and the immune anti-tumor response in order to find the perfect equilibrium, windows to give OVs and consequently the best way to fight cancer. To achieve this goal, new investigations are ongoing in order to design viruses that could stimulate specific immune populations thereby exploiting the true potential of the innate weapon. The final aim of all these studies is to stimulate the immune system to give rise to the correct response, avoiding aberrant inflammation that could result in a risk for patients. Moreover, recent studies have demonstrated that the true potential of OVT is in combination with classical treatments and other new therapies. Next generation studies should be focused on achieving these two aspects: making the viruses safer and more powerful and allowing them to work in synergy with other compounds.

## Author Contributions

YW provided the conception of the article; GM, AH, and YW designed the article; GM and AH drafted the article while YW made critical revisions related to important intellectual content of the manuscript and approved the final version of the article to be published with Nick Lemoine.

## Conflict of Interest Statement

The authors declare that the research was conducted in the absence of any commercial or financial relationships that could be construed as a potential conflict of interest.
